# Generalization of the small-world effect on a model approaching the Erdős–Rényi random graph

**DOI:** 10.1038/s41598-019-45576-3

**Published:** 2019-06-25

**Authors:** Benjamin F. Maier

**Affiliations:** 1 Robert Koch-Institute, Nordufer 20, D-13353 Berlin, Germany; 2 Department of Physics, Humboldt-University of Berlin, Newtonstraße 15, D-12489 Berlin, Germany

**Keywords:** Complex networks, Systems analysis

## Abstract

The famous Watts–Strogatz (WS) small-world network model does not approach the Erdős–Rényi (ER) random graph model in the limit of total randomization which can lead to confusion and complicates certain analyses. In this paper we discuss a simple alternative which was first introduced by Song and Wang, where instead of rewiring, edges are drawn between pairs of nodes with a distance-based connection probability. We show that this model is simpler to analyze, approaches the true ER random graph model in the completely randomized limit, and demonstrate that the WS model and the alternative model may yield different quantitative results using the example of a random walk temporal observable. An efficient sampling algorithm for the alternative model is proposed. Analytic results regarding the degree distribution, degree variance, number of two-stars per node, number of triangles per node, clustering coefficient, and random walk mixing time are presented. Subsequently, the small-world effect is illustrated by showing that the clustering coefficient decreases much slower than an upper bound on the message delivery time with increasing long-range connection probability which generalizes the small-world effect from informed searches to random search strategies. Due to its accessibility for analytic evaluations, we propose that this modified model should be used as an alternative reference model for studying the influence of small-world topologies on dynamic systems as well as a simple model to introduce numerous topics when teaching network science.

## Introduction

When Watts and Strogatz published their 1998 paper “Collective dynamics of’small-world’ networks”^1^, it had a phenomenal influence on the field of complex systems and was one of the defining studies for the following success of network science to emerge as an interdisciplinary field. Not only was it the first of a succession of studies^2–5^ trying to explain the small-world effect as based on Milgrim’s “six degrees of separation” experiment^6^, it introduced a simple and intuitive network model which had, at its core, the defining properties to obtain a “complex” system. Starting with a regular, locally connected structure, a rewiring process introduces long-range edges until it ends in a completely randomized state, thus interpolating between two well-studied physical systems: a crystal and disorder. For even small amounts of rewired contacts, the probability that two neighbors are connected (typically large in social networks^7^) hardly changes, while almost immediately, short paths between individual nodes appear, explaining how social networks can be both: highly clustered but with a small amount of necessary steps to reach one node from another.

This network model, based on rewiring edges, is widely used in the network literature, often to explore the influence of the small-world effect on the outcome of dynamic processes taking their course on the network. One important feature of the model is that the mean degree, i.e. the average number of nodes a node is connected to, is constant, which is a first-order control parameter for a variety of dynamic systems based on, e.g. random walks or epidemic spreading^8^. Within this kind of research, people sometimes argue that one limit of the rewiring process reproduces *the* random graph, i.e. the Erdős–Rényi $G(N,p)$ random graph model (as, for instance, in refs.^9–11^), where *N* is the number of nodes and *p* is the probability that any two nodes are connected. However, due to the model’s definition, the maximally randomized Watts-Strogatz model does *not* actually equal the Erdős–Rényi model. More often, references to the disorded limit do not directly mention the Erdős–Rényi model as a limit but are ambiguous in their wording and thus easily misinterpretable, see e.g. refs.^12–18^. Likewise, the original rewiring procedure, where each node rewires each of its edges to its *rightmost* neighbors with probability *p*_*r*_ is similarly misinterpreted, to mean just rewiring any edge. While the differences or slight variations of the model might not be influential for some dynamics, in others they can cause clear deviations from expected results in random graphs (see e.g. Sec. “Model definitions and differences”), thus potentially leading to confusion or faulty interpretations. The model is part of virtually every network science curriculum, however, actually calculating the clustering coefficient, the degree distribution or the small-world effect with pen and paper is often omitted since these observables or effects are complicated to evaluate. We argue that a model where those properties can be easily evaluated without the aid of a computer and actually reproduce formerly derived results from the Erdős–Rényi model might keep students more engaged and trained to calculate properties of other network models.

A model which solves the problems discussed above has been introduced by Song and Wang^19^. Within their study, they showed that sampling edges from a distance-based connection probability eases the evaluations of e.g. the degree distribution and the clustering coefficient. In this paper, we reformulate and discuss this modified model, propose an efficient sampling algorithm, extend the evaluation of degree distribution and clustering coefficient to other network properties, and show how it can be used to explain the small-world effect analytically by comparing the clustering coefficient to an upper bound of the message delivery time. Since the shortest path length equals the delivery time of an optimal search process between two nodes, the result presented here generalizes the small-world effect to random search strategies.

## Results

### Model definitions and differences

In the original model *N* nodes are positioned equidistantly on a ring and subsequently *locally* connected, i.e. connected to nodes in their vicinity with maximal lattice distance $d\le k$/2 where *k* is an even positive integer. In this state each node has degree *k*, where “degree” refers to the number of neighbors of a node. For the rewiring process, each node rewires its connections to its *k*/2 rightmost neighbors to any other node in the network with probability *p*_*r*_. It is easy to see that at the randomized limit of ${p}_{r}=1$, each node has minimum degree *k*/2. Furthermore, an original edge connected to a node *u* has been rewired and can only exist if it is reproduced by another rewiring event based on its corresponding rightmost neighbor. This implies that at ${p}_{r}=1$ an original edge exists with probability 1/$(N-1)$. Both these properties lead to conceptual deviations from the Erdős–Rényi model in which each edge exists with probability ${p}_{{\rm{ER}}}=k$/$(N-1)$ and nodes may have degree <*k*/2.

In a variant of the modified model by Song and Wang^19^ presented in the following, edges posses an inherent probability to exist, which varies for *short*-*range* (*S*) and *long*-*range* (*L*) contacts. A potential contact between nodes (*i*, *j*) is considered to be short-ranged if their distance in periodic boundary conditions is $d(i,j)\le k$/2; it exists with probability *p*_*S*_. It is considered long-range if $d(i,j) > k$/2 and exists with probability *p*_*L*_. The distance is computed as $d(i,j)=\,{\rm{\min }}(|j-i|,N-|j-i|)$. In short, two nodes with lattice distance *d* are connected with probability

$${p}_{d}={\textstyle \{}\begin{array}{cc}{p}_{S}, & {\rm{i}}{\rm{f}}\,d\le k/2,\\ {p}_{L}, & {\rm{o}}{\rm{t}}{\rm{h}}{\rm{e}}{\rm{r}}{\rm{w}}{\rm{i}}{\rm{s}}{\rm{e}}.\end{array}$$

Hence, if ${p}_{S}=1$ and ${p}_{L}=0$, the model produces a structure which is equal to the original model’s starting point, a one-dimensional *k*-nearest neighbor lattice. On the other hand, if ${p}_{S}={p}_{L}\equiv p$, each edge exists with probability *p* and hence the model reproduces the $G(N,p)$ random graph. We can fix the mean degree by noticing that it is composed of a short-range degree $\langle {k}_{S}\rangle$ and a long-range degree $\langle {k}_{L}\rangle$. Each node has *k* potential short-range neighbors and $N-1-k$ potential long-range neighbors. Thus, its expected degree is

1 $${p}_{S}k+{p}_{L}(N-1-k)=\langle {k}_{S}\rangle +\langle {k}_{L}\rangle =\langle k\rangle \equiv k.$$

To keep the mean degree constant, we introduce a control parameter *β* which controls the trade-off of connection probability in the short- and long-range regimes such that ${p}_{L}=\beta {p}_{S}$. Note that at $\beta =0$, we have ${p}_{L}=0$ such that from Eq. (1) it follows that ${p}_{S}=1$ while at $\beta =1$ we find ${p}_{L}={p}_{S}\equiv p$. In order for the mean degree to be constant, Eq. (1) yields the distance-based probabilities

2a $${p}_{S}(\beta )=\frac{1}{1+\beta (N-1-k)/k},$$

2b $${p}_{L}(\beta )=\frac{\beta }{1+\beta (N-1-k)/k}=\beta {p}_{S}(\beta ).$$

**Figure 1:**
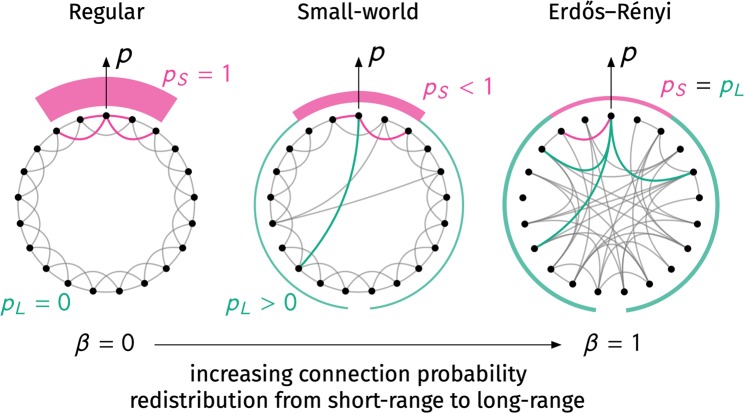
Schematic representation of the alternative small-world model as introduced in^19^ and discussed in this paper. Much like in the original model, we start with *N* nodes placed equidistantly on a ring. However, instead of rewiring, each pair of nodes is connected with distance-based probability *p*_*d*_ where *d* is their minimal distance on the ring. Within distance $d\le k$/2, nodes are connected with short-range probability *p*_*S*_. For larger distances, nodes are connected with long-range probability ${p}_{L}=\beta {p}_{S}$. With increasing redistribution parameter $0\le \beta \le 1$ connection probability is redistributed from the short-range regime to the long-range regime while the mean degree *k* i﻿”Acknowledgements” on page 9s kept constant. Hence at $\beta =0$ the short-range probability is unity while the long-range probability is zero which produces a *k*-nearest neighbor lattice. With increasing *β*, long-range “short-cuts” become more probable until at $\beta =1$ both connection probabilities are equal and thus the model becomes equal to the Erdős–Rényi model.

The short-range node degree *k*_*S*_ follows a binomial distribution ${\mathcal B} (k,{p}_{S})$ and the long-range node degree *k*_*L*_ follows a binomial distribution ${\mathcal B} (N-1-k,{p}_{L})$ where ${\mathcal B} (n,p)$ has probability mass function $${f}_{k}(n,p)=(\begin{array}{c}n\\ k\end{array}){(1-p)}^{n-k}{p}^{k}$$. A schematic explanation of the model is shown in Fig. 1. A simple network generation algorithm is given as follows. Each node $0\le u\le N-1$ connects to each of its *k*/2 rightmost short-range neighbors with probability *p*_*S*_. Afterwards, *m*_*L*_ long-range edges are drawn, where *m*_*L*_ follows ${\mathcal B} (N(N-1-k)/2,{p}_{L})$. For each long-range edge one chooses a source node *u* uniform at random from $[0,N-1]$. This node is then connected to a long-range neighbor $v=(u+k/2+z)\,{\rm{mod}}\,N$ where the integer *z* is drawn uniform at random from the interval $[1,N-k-1]$. If an already existing edge was chosen, repeat the procedure for this long-range edge. This algorithm has complexity ${\mathscr{O}}(Nk+\langle {m}_{L}\rangle )$ for sparse networks. Open source implementations of the algorithm are available as C++/Python packages^20,21^.

**Figure 2:**
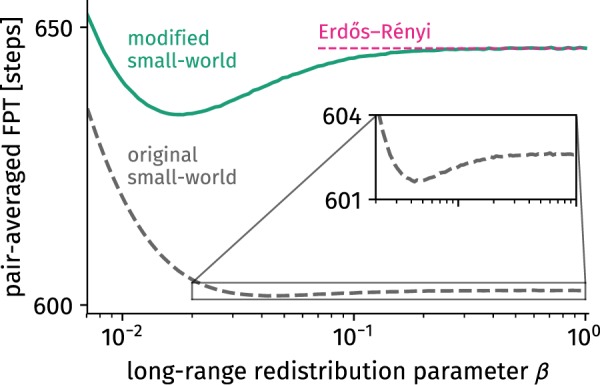
The pair-averaged first passage time (PAFPT) of a discrete-time random walk process is an example of a network observable differing from the corresponding result of the Erdős–Rényi model in the limit of $\beta =1$. In contrast, the result from the modified model described in Sec. “Model definitions and differences” approaches the desired limit. Inset: A minimum in the PAFPT emerges in both the modified as well as the original model, an effect explained in ref.^26^.

As the original model is widely used, we aim to highlight potential consequences for the misinterpretation of the original model’s randomized limit in the following and compare it to the corresponding results of the alternative model, which does approach the Erdős–Rényi model. To this end it is first necessary to map the control parameters of the two models in an appropriate manner such that they will be in similar states when varying the parameters. We note that for small *β* the short-range connection probability *p*_*S*_ should be approximately equal to the probability that an edge has *not* been rewired $1-{p}_{r}$ in the original model. To ensure that ${p}_{r}=1$ for $\beta =1$ we set ${p}_{r}=[1-{p}_{S}]$/$[1-{p}_{{\rm{ER}}}]$. In order to compare the structural consequences on dynamic observables of both models, we will compute a temporal observable of a discrete-time random walk process as an example, a process defined as follows: At each discrete time step a walker residing on a node *u* chooses to jump to any of *u*’s neighbors with uniform probability, repeated indefinitely. Random walks are widely applied to model spreading and search processes in physics, biology, and computer science^7,8,22–25^. Within this context, the mean first passage time ${\tau }_{vu}$ is the expected number of steps a random walker needs to traverse to node $v$ when it started at node *u* which therefore can be interpreted as an upper bound for any search process^26^. In contrast, the shortest path length between two nodes is the search time for an optimal search process. Based on the mean first passage time, the pair-averaged first passage time $\tau ={(N(N-1))}^{-1}{\sum }_{v=1}^{N}\,{\sum }_{u\ne v}^{N}\,{\tau }_{uv}$ acts as a coarse-grained estimation of how fast a random search process can be conducted between any two nodes of a particular network. We computed the pair-averaged first passage time for small-world networks of $N=512$ nodes and mean degree $k=10$. The control parameters of both the alternative and the original model were varied (*β* and ${p}_{r}=(1-{p}_{S}(\beta ))$/$(1-{p}_{{\rm{ER}}})$, respectively). For each value of *β* we built the average of the pair-averaged first passage time over the largest component of 10,000 independent network realizations. For each realization, the mean first passage times between all pairs of nodes of the largest component were computed using Eq. (14) in ref.^27^. The results shown in Fig. 2 imply that, indeed, the difference between both models can be of significance, reaching values of a relative difference of up to ≈7% in the randomized limit. This difference is induced by the fact that in the original model, each node has a minimum degree of *k*/2 whereas in the modified model nodes of smaller degree may exist^26^.

The emergence of such a difference is an indicator for the relevance of the alternative model for studying the influence of small-world topologies on the outcome of dynamic systems – the alternative model is suited to compare its implications to the implications of a known model, the Erdős–Rényi graph.

### Network properties of the alternative model

We begin our discussion of the network properties with the degree variance, which is important to quantify the heterogeneity of nodes in a network based on their connectivity: It has been shown that increased degree variance is increasing the risk of endemicity of diseases on a network^8^. Furthermore, the degree variance plays an important role to estimate the average arrival time of random walks^26^. Because in the alternative small-world model the node degree is given as the superposition of short-range and long-range degree, the degree variance can be simply computed as

$$\begin{array}{rcl}{\rm{Var}}[k] & = & {\rm{Var}}[{k}_{S}]+{\rm{Var}}[{k}_{L}]\\  & = & k{p}_{S}(1-{p}_{S})+(N-1-k){p}_{L}(1-{p}_{L}).\end{array}$$

**Figure 3:**
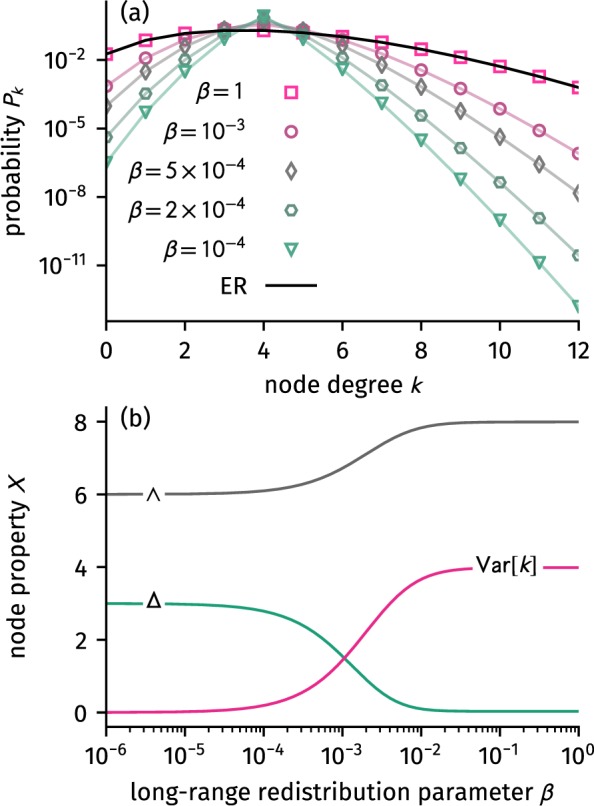
Analytic results for (**a**) degree distribution Eq. (3) and (**b**), expected number of two-stars per node $\wedge$, expected number of triangles per node Δ (Eq. (4)), and node degree variance as given in the main text. While both degree variance and number of two-stars increase with increasing long-range redistribution parameter *β*, the number of triangles decreases. The results shown here were computed for $N=1001$ and $k=4$.

For increasing *β* both short-range and long-range variances increase, as well, such that the degree variance is an increasing function of *β*, as shown in Fig. 3b. The full degree distribution is computable by noting that any node degree is ${k}_{i}={k}_{S,i}+{k}_{L,i}$, such that its distribution is given by the convolution

3 $$\begin{array}{ccc}{p}_{k^{\prime} } & = & \sum _{{k}_{S}=0}^{{\rm{\infty }}}\,\sum _{{k}_{L}=0}^{{\rm{\infty }}}\,{f}_{{k}_{S}}(k,{p}_{S}){f}_{{k}_{L}}(N-1-k,{p}_{L}){\delta }_{k^{\prime} ,({k}_{S}+{k}_{L})}\\  & = & \sum _{{k}_{S}=0}^{min(k^{\prime} ,k)}\,(\begin{array}{c}k\\ {k}_{S}\end{array})\,(\begin{array}{c}N-1-k\\ k^{\prime} -{k}_{S}\end{array})\,{(1-{p}_{S})}^{k-{k}_{S}}\\  &  & \,\,\times \,{p}_{S}^{{k}_{S}}{(1-{p}_{L})}^{N-1-k-k^{\prime} +{k}_{S}}{p}_{L}^{k^{\prime} -{k}_{S}},\end{array}$$

which is similar to the result derived in ref.^19^ and is shown in Fig. 3a. Note that in the derivation above we used Kronecker’s delta ${\delta }_{ij}=0$ if $i\ne j$ and ${\delta }_{ij}=1$ otherwise. Both the results of the degree variance and the degree distribution highlight the simplicity of the alternative model, which allows for a simple analytical evaluation as compared to more complicated derivations in the original model based on rewiring^5^.

While there exist multiple similar definitions, the clustering coefficient is usually reflecting the probability of triadic closure: Given a structure where a node *i* is connected to nodes *v* and *u*, the clustering coefficient is the probability that *u* and *v* are connected, as well. Using the network’s $(N\times N)$-sized adjacency matrix ${A}_{ij}=1$ if nodes *i* and *j* are connected and ${A}_{ij}=0$ otherwise, we therefore define the global clustering coefficient as the conditional probability

$$C=P[{A}_{iu}{A}_{uv}{A}_{vi}=1|{A}_{iu}{A}_{iv}=1]=\frac{\langle {A}_{iu}{A}_{uv}{A}_{vi}\rangle }{\langle {A}_{iu}{A}_{iv}\rangle }\equiv \frac{{\rm{\Delta }}}{\wedge },$$

similar to the definition in ref.^19^. We will, however, derive the final result using a more geometric approach in the following. The probability $\wedge =\langle {A}_{iu}{A}_{iv}\rangle$ is the expected number of two-stars per node (a structure where node *i* is connected to both a node *u* and a node *v*). To evaluate this quantity one observes that a node of degree *k*_*v*_ is part of $(1/2){k}_{v}({k}_{v}-1)$ two-stars. Therefore, it is given as $\wedge =(1/2)\,[{\rm{Var}}[k]+k(k-1)]$. It hence qualitatively follows the behavior of the degree variance as illustrated in Fig. 3b.

In order to find the expected number of triangles per node we recognize that every node is statistically equivalent. Thus, without loss of generality, we compute the number of triangles per node $i=1$ as the sum over all possible remaining node pairs considering their distance-based connection probability as

4 $$\begin{array}{rcl}{\rm{\Delta }} & = & \sum _{u=2}^{N-1}\,\sum _{v=u+1}^{N}\,{p}_{d(u,1)}{p}_{d(v,1)}{p}_{d(u,v)}\\  & = & F{p}_{S}^{3}+G{p}_{S}^{2}{p}_{L}+H{p}_{S}{p}_{L}^{2}+I{p}_{L}^{3}.\end{array}$$

**Figure 4:**
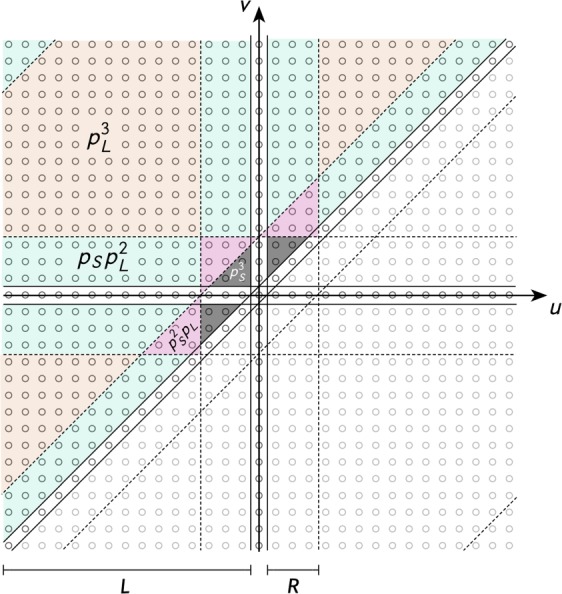
Evaluation of the areas of summation to find the expected number of triangles per node Δ for odd numbers of nodes *N* as per Eq. (4). Note that here, the sum has been shifted to be ${\sum }_{u=-(N-1)/2+1}^{(N-1)/2}\,{\sum }_{v=u+1}^{(N-1)/2}\,(\,\cdot \,)$ such that *u* and *v* are equal to their lattice distance to a focal node 0.

Here, *F*, *G*, *H*, and *I* are the areas of summation highlighted in Fig. 4 where three (grey), two (pink), one (green), and no (orange) node pairs are of short-range distance, respectively. Considering the case of odd numbers of *N* one may shift the summations to run from lattice distance −*N*/2 to distance *N*/2 around a focal node at $d=0$ such that by marking the conditions for short-range connections, finding the respective areas reduces to a geometrical exercise. By defining the lengths $L=(N-1)$/2 and $R=k$/2 as marked in Fig. 4, one first finds the useful unit of a short-short-long-range area as the triangle $T=({R}^{2}-R)$/$2+R$ (marked as pink in Fig. 4). Then, the areas of summation are given as

$$\begin{array}{rcl}F & = & 3(T-R)=(3k/8)\,(k-2)\\ G & = & 3T=(3k/8)\,(k+2)\\ H & = & 2((L-R)R-T)+T+2(L-R)R+2((L-1)R-T)\\  & = & (k/8)\,(12N-26-11k)\\ I & = & {(L-R)}^{2}-2((L-1)R-T)-(L-R)+{(L-R)}^{2}-T\\  & = & (1/8)\,[5{k}^{2}-k(12N-26)+4({N}^{2}-3N+2)].\end{array}$$

The expected number of triangles Eq. (4) consequently decreases with increasing *β*, as expected and as shown in Fig. 3b. Considering Eq. (2), the exact value of the clustering coefficient is then given by

5 $$C(\beta )={p}_{S}^{3}\times \frac{F+G\beta +H{\beta }^{2}+I{\beta }^{3}}{(1/2){\rm{Var}}[k]+k(k-1)}.$$

In the respective limits we find

$$\begin{array}{rcl}C(\beta =0) & = & \frac{3(k-2)}{4(k-1)}\\ C(\beta =1) & = & \frac{{\sum }_{u=2}^{N-1}\,{\sum }_{v=u+1}^{N}\,{p}^{3}}{{\sum }_{u=2}^{N-1}\,{\sum }_{v=u+1}^{N}\,{p}^{2}}=p,\end{array}$$

which are the expected results for both the *k*-nearest neighbor lattice as well as the Erdős–Rényi graph. Further considering Eqs. (2) and (5) as well as noting that ${\rm{Var}}[k]\,(\beta \to 0)=0$, in the limit of small long-range redistribution one finds

6 $$\frac{C(\beta \ll 1)}{C(0)}\approx {p}_{S}^{3}=1-3\beta \frac{N-k-1}{k}+{\mathscr{O}}({\beta }^{2})$$

which will be of importance for quantifiying the small-world effect in the following.

### Small-world effect

In the original model, the small-world effect was illustrated by comparing the clustering coefficient to the average shortest path length of networks. While random networks have short path lengths, they possess low clustering, on the other hand regular networks are highly clustered, while nodes are, on average, quite distant from one another. With rewiring only a short amount of edges of an ordered network it was shown that shorter paths appear immediately while high clustering preserves, explaining the small-world effect. It has further been argued that algorithmic searches requiring local information are necessary to identify these short paths^2,3^. However, in situations where searches are less targeted and follow rather diffusive dynamics such as epidemic spreading over air traffic^28^ or synchronization in oscillators^8^, the role of the mean shortest path length becomes less prominent. Rather, random walk relaxation and passage times are the important observables characterizing these dynamics, specifically to predict the arrival time of a disease or the likelihood of global synchronization. Therefore, we will take an approach focusing on random walks in the following.

One of the purposes of the original model was to explain the Milgram small-world experiment^6^ where participants had to mail letters to strangers by mailing them to a person they did know and instruct them to pass the letter further. In the following we will illustrate the small-world effect by showing that an upper bound for the delivery time of those messages decreases much faster than the clustering coefficient with increasing probability of long-range edges. Since this upper bound of a random search also bounds the mean shortest-path length which is the equivalent to the arrival time of a maximally informed search, the following result generalizes the small-world effect to random dynamics.

Considering completely uninformed individuals, the mailing process is modeled as a random walk process where the random walkers correspond to the letters to be sent to recipients. At each integer time step *t*, the letter resides on a node *u*. Subsequently, one of *u*’s neighbors *v* is chosen uniform at random as the next recipient of the message. At the next time step $t+1$ the letter then resides at node *v*. This process is repeated indefinitely and is governed by the master equation ${\phi }_{v}(t)={\sum }_{u=1}^{N}\,({A}_{vu}/{k}_{u}){\phi }_{u}(t-1)$ where ${\phi }_{v}(t)$ is the probability that the letter is on node *v* at time *t* and ${W}_{vu}={A}_{vu}$/*k*_*u*_ is the probability that the letter is sent from node *u* to node *v*. Instead of generating adjacency matrices and averaging over the results of their corresponding transition matrices we will compute an average medium matrix where each edge in the network is replaced by the probability of this edge existing such that ${W}_{vu}^{{\rm{avg}}}=\langle {A}_{vu}\rangle$/$k={p}_{d(v,u)}$/*k*. One can show that the time scale with which the equilibrium distribution is approached on this average medium network is given by the eigenvalue gap of the transition matrix ${W}_{vu}^{{\rm{avg}}}$ as ${t}_{{\rm{mix}}}^{-1}=1-{\omega }_{1}$ where ${\omega }_{0}=1$ is the largest eigenvalue and ${\omega }_{1}$ is the second largest eigenvalue^29^. The average medium transition matrix ${W}_{vu}^{{\rm{avg}}}$ is circulant based on the vector

$$w={k}^{-1}(0,\mathop{\underbrace{{p}_{S},\ldots ,{p}_{S}}}\limits_{k/2},\mathop{\underbrace{{p}_{L},\ldots ,{p}_{L}}}\limits_{N-1-k},\mathop{\underbrace{{p}_{S},\ldots ,{p}_{S}}}\limits_{k/2}).$$

In this case, the *j*-th eigenvalue of ${W}_{vu}^{{\rm{avg}}}$ is given as ${\omega }_{j}={\sum }_{v=1}^{N}\,{w}_{v}\,\exp (i2\pi v/N)$ such that the second largest eigenvalue can be easily computed as ${\omega }_{1}={p}_{S}{\rm{\Gamma }}/k-{p}_{L}(1+{\rm{\Gamma }})/k$ where ${\rm{\Gamma }}$ = $2\,{\sum }_{j=1}^{k/2}\,\cos (2\pi j/N)$ = $k-{(\pi /N)}^{2}k(k/2+1)(k+1)/3+{\mathscr{O}}({N}^{-4})$ which yields the mixing time

7 $${t}_{{\rm{m}}{\rm{i}}{\rm{x}}}(\beta )={[1-\frac{{\rm{\Gamma }}-\beta (1+{\rm{\Gamma }})}{k+\beta (N-1-k)}]}^{-1}.$$

**Figure 5:**
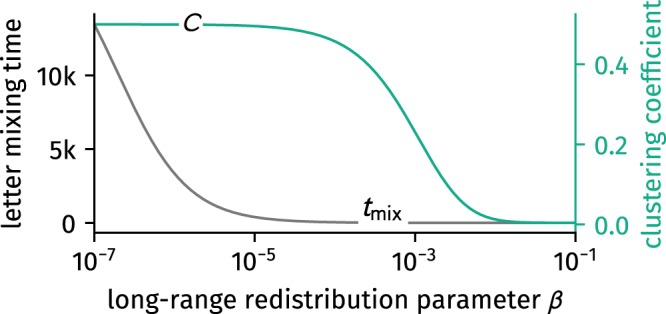
The small-world effect as illustrated by the observables computed analytically in this paper. The random walk message delivering mixing time Eq. (7) as an upper bound of the targeted search message delivering mixing time decreases rapidly with the introduction of long-range links while clustering Eq. (5) preserves. Displayed here are results for $N=1001$ and $k=4$.

In Fig. 5 we show how both clustering coefficient and mixing time decrease with increasing long-range redistribution parameter *β*. In the limits we find the expected results from *k*-regular networks and an average medium approximation of the Erdős–Rényi graph

$$\begin{array}{ccc}{t}_{{\rm{m}}{\rm{i}}{\rm{x}}}(\beta =0) & = & {[1-\frac{{\rm{\Gamma }}}{k}]}^{-1}\mathop{\to }\limits^{N\gg k/2}\frac{{N}^{2}}{{\pi }^{2}}\frac{3}{(k/2+1)\,(k+1)},\\ {t}_{{\rm{m}}{\rm{i}}{\rm{x}}}(\beta =1) & = & {[1-\frac{1}{N-1}]}^{-1}=1-\frac{1}{N}.\end{array}$$

This implies that for small long-range redistributions the relative mixing time decreases as

8 $$\frac{{t}_{{\rm{m}}{\rm{i}}{\rm{x}}}(\beta )}{{t}_{{\rm{m}}{\rm{i}}{\rm{x}}}(0)}=1-\beta (\frac{3{N}^{3}}{{\pi }^{2}(k/2+1)\,(k+1)k}-\frac{N}{k}+\frac{1}{k})+{\mathscr{O}}({\beta }^{2}).$$

Comparing Eqs. (6) and (8), one can easily see that for small *β* the rate with which the mixing time decreases is of order *N*^3^ while the rate with which the clustering coefficient decreases is of order *N*, which is a difference of two orders of magnitude. This shows that even with a small amount of long-range connection probability, the delivery time of randomly passed messages declines rapidly while clustering is still preserved. Since an optimal search strategy identifies the shortest path between two nodes and the original small-world effect was shown for those shortest paths, this result generalizes the small-world effect to random search strategies.

## Discussion

We discussed an alternative small-world network model first introduced in ref.^19^, which approaches the Erdős–Rényi random graph model in the limit of maximum disorder and showed that the original small-world network model does not. Within this model, instead of rewiring edges, long-range contacts are introduced by redistributing connection probability from short-range to long-range potential neighbors while keeping the mean degree constant. Constructing small-world networks in this way allows for a thorough analytical analysis of network properties such as the degree distribution, the degree variance, the average number of two-stars, the average number of triangles, and the clustering coefficient. An upper bound of the message deliviery time can be computed using an average medium approximation. We showed that for a small amount of redistributed long-range connection probability the clustering coefficient decreases with a rate proportional to the number of nodes *N* while the upper bound of the delivery time decreases with a rate of order *N*^3^, hence illustrating how social networks can have both high clustering as well as a favorable topology to efficiently forward messages to unknown recipients, even if the search strategies are purely random, as they might be in diffusive contexts such as epidemic spreading in air traffic or synchronization of oscillators.

In the following we will discuss the modified model’s applicability to teach network concepts. As network theory curricula typically introduce Erdős–Rényi random graphs early on as one of the first network models, the concept of drawing edges with a certain probability is known to students. We argue that extending this concept to draw edges from two categories (short-range and long-range) with two connection probabilities is a natural way to extend this formalism on a path to more complicated models. Based on the derivation of the degree distribution of the random graph one can easily comment on the distribution of random variables’ superposition and derive the degree distribution of the small-world model. Subsequently, similarly to the clustering coefficient computable in the Erdős–Rényi model, the clustering coefficient of the modified small-world model can be computed as the conditional probability that two nodes are connected given that they are neighbors of a focal node, in contrast to the local clustering coefficient in the original model. This further allows for the introduction of an average medium where each edge is replaced by the probability that it exists. Consequently using this average medium approximation one can use the modified model to introduce the random walk formalism and show how to evaluate its mixing time to arrive at the small-world effect based on the message delivery time and the Milgram small-world experiment (with a careful discussion of its flaws). We furthermore argue that the more simplistic picture of drawn instead of rewired edges is more intuitive. Instead of an individual explicitly deciding to change one of its short-range contacts to a long-range contact, there is an inherent probability to be connected to “near” nodes as well as a smaller probability to be connected to nodes “further away”.

Finally, we suggest the modified model to be used as an alternative to the original model when studying the influence of the small-world effect on dynamic systems, since the modified model truly interpolates between two well-studied systems, a nearest-neighbor lattice and the Erdős–Rényi model. It therefore allows for simpler and more reliable comparisons of results and potentially offers more insight to other dynamics due to its analytical accessibility.

## Materials

The network sampling algorithm described in Sec. “Model definitions and differences” is implemented for Python and C++ and available for download^20,21^. Additionally, several Python functions to compute the model’s network properties as well as the average medium mixing time are implemented in ref.^21^.

## Data Availability

No datasets were generated or analysed during the current study.
