# Publisher Correction: Generalization of the small-world effect on a model approaching the Erdős–Rényi random graph

**DOI:** 10.1038/s41598-020-60474-9

**Published:** 2020-02-21

**Authors:** Benjamin F. Maier

**Affiliations:** 1 Robert Koch-Institute, Nordufer 20, D-13353 Berlin, Germany; 2 Department of Physics, Humboldt-University of Berlin, Newtonstraße 15, D-12489 Berlin, Germany

Correction to: *Scientific Reports* 10.1038/s41598-019-45576-3, published online 25 June 2019

The original version of this Article contained an error in the legend of Figure 1.

“Schematic representation of the alternative small-world model as introduced in^19^ and discussed in this paper. Much like in the original model, we start with $N$ nodes placed equidistantly on a ring. However, instead of rewiring, each pair of nodes is connected with distance-based probability ${p}_{d}$ where $d$ is their minimal distance on the ring. Within distance $d\le k/2$, nodes are connected with short-range probability ${p}_{S}$. For larger distances, nodes are connected with long-range probability ${p}_{L}={\beta p}_{S}$. With increasing redistribution parameter $0\le \beta \le 1$ connection probability is redistributed from the short-range regime to the long-range regime while the mean degree $k$ i﻿“Acknowledgements” on page 9 s kept constant. Hence at $\beta =0$ the short-range probability is unity while the long-range probability is zero which produces a $k$-nearest neighbor lattice. With increasing $\beta$, long-range “short-cuts” become more probable until at $\beta =1$ both connection probabilities are equal and thus the model becomes equal to the Erdős–Rényi model.”

now reads:

“Schematic representation of the alternative small-world model as introduced in^19^ and discussed in this paper. Much like in the original model, we start with $N$ nodes placed equidistantly on a ring. However, instead of rewiring, each pair of nodes is connected with distance-based probability ${p}_{d}$ where $d$ is their minimal distance on the ring. Within distance $d\le k/2$, nodes are connected with short-range probability ${p}_{S}$. For larger distances, nodes are connected with long-range probability ${p}_{L}={\beta p}_{S}$. With increasing redistribution parameter $0\le \beta \le 1$ connection probability is redistributed from the short-range regime to the long-range regime while the mean degree $k$ i﻿s kept constant. Hence at $\beta =0$ the short-range probability is unity while the long-range probability is zero which produces a $k$-nearest neighbor lattice. With increasing $\beta$, long-range “short-cuts” become more probable until at $\beta =1$ both connection probabilities are equal and thus the model becomes equal to the Erdős–Rényi model.”

This error has now been corrected in the PDF and HTML versions of the Article.

